# Supplementary figures and images for: The impact of COVID-19 on trips to urban amenities: Examining travel behavior changes in Somerville, MA
Source: PLoS One. 2021 Sep 1;16(9):e0252794. doi: 10.1371/journal.pone.0252794 (PMC8409662; doi:10.1371/journal.pone.0252794)

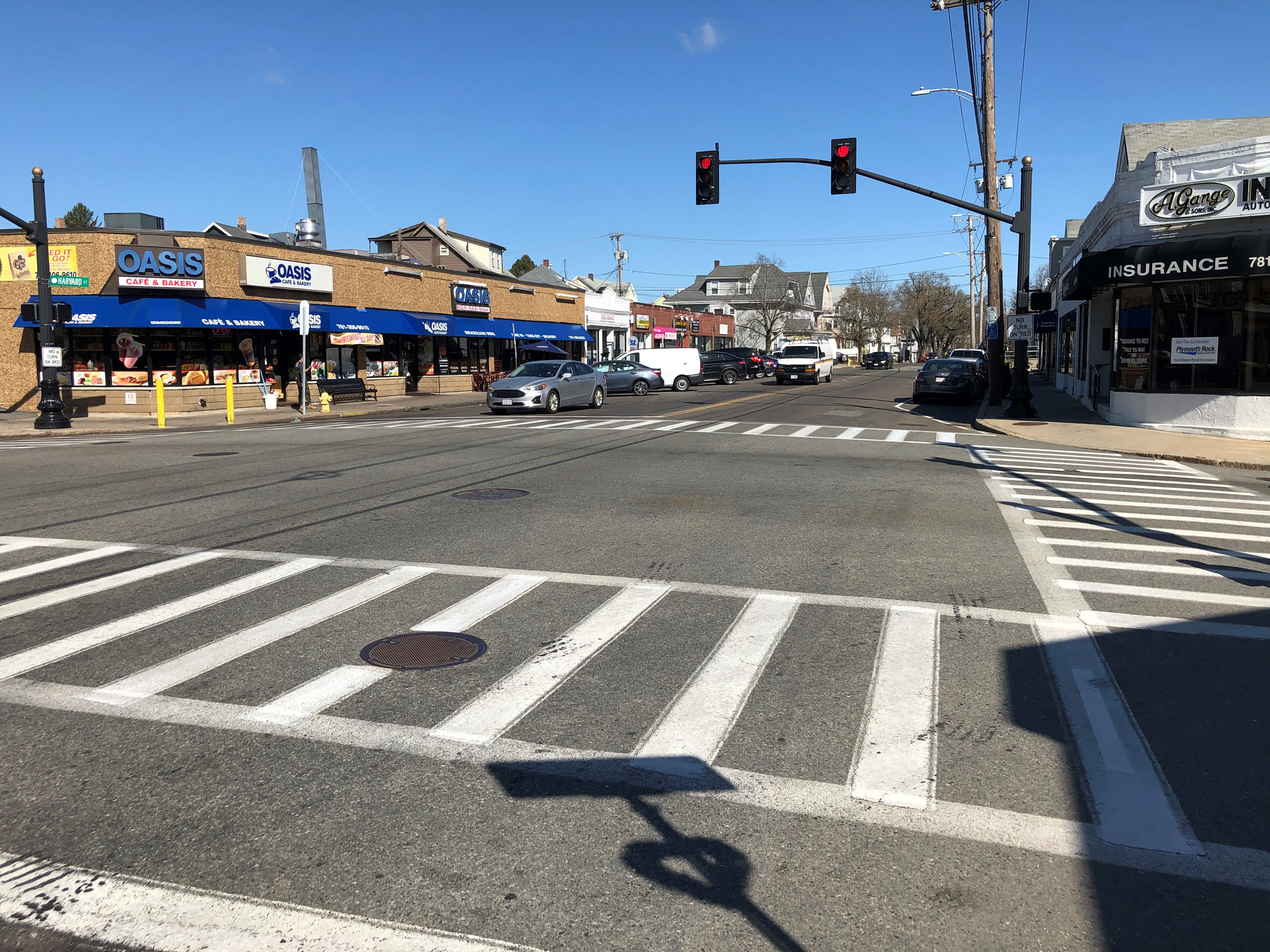

Supplement: S1 Fig — Amenity cluster on Main Street in South Medford, featuring a popular Brazilian buffer restaurant “Oasis”, which featured a 198% growth in visits between April-December 2020, compared to the same months the year before. (Image source: authors). (TIF) [file pone.0252794.s002.tif]
