# Supplementary material for: The impact of COVID-19 on trips to urban amenities: Examining travel behavior changes in Somerville, MA
Source: PLoS One. 2021 Sep 1;16(9):e0252794. doi: 10.1371/journal.pone.0252794 (PMC8409662; doi:10.1371/journal.pone.0252794)
Supplement: S1 File — (DOCX) [file pone.0252794.s003.docx]

**S1 File. Supplementary materials**

1. **City of Somerville Shutdown Timeline.**

As part of the City of Somerville’s unprecedented shutdown order of May 24th, 2020, only essential food and beverage businesses (e.g. supermarkets, restaurants), health care businesses (e.g. pharmacies), and construction businesses (e.g. home improvement stores) could stay open for business. Restaurants were limited to take-out and deliveries only. These restrictions lasted for almost two months. On May 18^th^ 2020, construction and religious activities could resume. On May 25th, retailers (other than essential retailers) were allowed to offer curb-side pickup. Phase one of reopening started on June 1st, when a number of more business openings followed: offices, hair and beauty salons, car washes, manufacturing and science-oriented businesses, all with reduced capacity. Phase two of reopening in Somerville commenced a week later, on June 8th, allowing hotels, in-store retailers, childcare establishments, car dealerships and funeral homes to resume business. Most of the businesses that could reopen faced new regulations about hygiene provision, social distancing, and maximum customer capacity. A limited phase three reopening commenced on September 8^th^, allowing movie theaters and outdoor performance venues, museums, cultural and historical sites, fitness centers and health clubs, professional sports teams, and other indoor recreational activities with low potential for contact to reopen. Thus, between March 24th and June 8th a large proportion of businesses had to be closed or limited to take-out and curbside pick-up only. Between June 8^th^ and September 8^th^, movie theaters and outdoor performance venues, museums, cultural and historical sites, fitness centers and health clubs remained closed. Phase three reopening were reversed to closed again between December 2020 and of January 2021.

1. **City of Somerville mobile phone penetration.**

Statista [49] estimates around 276 million smartphone users across the US in 2020, which constitutes 83% of the total population. According to 2019 data, Somerville’s median household income was similar to the national average ($64k in Somerville versus $63k nationally). However almost double (66%) of adults hold a college degree compared to the national average (33%) and the median age in Somerville is lower (31.2) than the national average (38.4 years).

1. **Variable Scaling for the Discrete Choice Model**

The dependent variable in the choice model represents the log of number of visits for each month. Among independent variables, distance (in km) is scaled by 1000. Number of establishments is scaled by 100. Percent large businesses is not scaled. Land values are scaled by 1000. Simpson entropy, which describes diversity of businesses, is not scaled [50]. Cluster area is scaled by 10^6. Cluster composition variables (% comparison, conveniences, F&B, entertainment, personal) are not scaled. Open space is scaled by 10^3.

|  | 2019 | | 2020 | |
| --- | --- | --- | --- | --- |
|  | Total number of trips to any cluster | Median distance traveled (meters) | Total number of trips to any cluster | Median distance traveled (meters) |
| Jan | 252,100 | 172 | 258,650 | 157 |
| Feb | 283,425 | 202 | 270,725 | 180 |
| Mar | 341,300 | 180 | 118,300 | 317 |
| Apr | 361,075 | 143 | 45,975 | 642 |
| May | 309,275 | 180 | 54,800 | 653 |
| Jun | 300,025 | 171 | 71,875 | 546 |
| Jul | 253,075 | 204 | 77,875 | 491 |
| Aug | 282,750 | 154 | 75,725 | 448 |
| Sep | 397,550 | 139 | 102,950 | 343 |
| Oct | 321,525 | 145 | 95,300 | 375 |
| Nov | 261,725 | 186 | 74,275 | 491 |
| Dec | 224,625 | 170 | 63,325 | 503 |

*Supp. Table 1. Total number of trips from CBGs and mean trip distance to amenity clusters during different months in 2019 and 2020.*

1. **Model Description**

Our implementation of the model assumes that visitors from CBGs *i* (where *i=*1*, n*) can choose to visit any of the retail clusters *k* (where *k=1, m*) around them. We assume that each amenity cluster $k$ is described by $g$ attributes and call the level of attributes $l$ *(where* $l=1,g)$*:*$Z_{lk}$. There are also travel costs involved in visiting each cluster. We symbolize the distance between each block group and amenity cluster along the shortest path as $T_{ik}$.

The overall utility ($U_{ik}$) that a visitor from a block group $i$ would derive from patronizing an amenity cluster $k$ can be shown as a linear function of cluster attributes $Z_{lk}$, travel costs $T_{ik}$, and a series of behavioral coefficients $\mu_{l}$that describe how people react to amenity cluster attributes $Z_{lk}$:

$$U_{ik}=\alpha T_{ik}+ \sum_{l=1}^{g} \mu_{l}Z_{lk}$$

(Supp. Equation 1)

In Equation 1, the first element represents the (dis)utility that visitors living in CBG $i$derive from travelling to cluster $k$, which depends on the distance $T_{ik}$between them. The coefficient $\alpha$ describes how much visitors dislike distance.

The last part of Equation 1 includes a summation of cluster attributes $Z_{lk}$and corresponding behavioral coefficients $\mu_{l}$that describe how visitors value each specific cluster attribute. When we multiply the behavioral coefficients $\mu_{l}$ with the cluster attribute $Z_{lk}$we get the estimated contribution of each attribute to the perceived cluster utility.

In a logistic choice framework, the probability that a visitor will visit each cluster is given as a ratio of exponents of that particular cluster’s utility divided by the sum of utilities of all clusters accessible to the visitor, including the cluster in question:

$$P_{ik}=\frac{e^{U_{ik}}}{\sum_{k=1}^{m} e^{U_{ik}}}$$

(Supp. Equation 2)

The expression in Equation 2 ensures that the probability to visit a particular cluster ranges between zero and one, with higher utility destinations obtaining a higher likelihood of visits than lower utility destinations. The total number of visitors estimated at each cluster depends simultaneously on the attributes ($Z_{lk}$) of the cluster relative to other available clusters, the location of the cluster relative to other clusters, as well as the spatial distribution of visitors. The total patronage ($S_{k}$) of a cluster $k$ is found by multiplying the total trips from each block group (to our destination set) by the probability of visits to that that cluster and summing across all block groups:

$$S_{k}=\sum_{i=1}^{n} P_{ik}N_{i}$$

(Supp. Equation 3)

The utility coefficients from Equation 1 can be empirically estimated using a linear regression model, by setting the dependent variable as ratio of the probability to visit each cluster relative to a chosen reference cluster in the area ($S_{ij1}/S_{ijk}$*)* and taking a log of this ratio. This produces regression coefficients that indicate how visits to a particular cluster compare to visits to the reference cluster. In our case, the regression coefficients illustrate how trips to a particular cluster *k=1* compare to not a specific reference cluster, but rather a “median” cluster available around each block group, which is characterized by median travel costs, a median route utility and median cluster attributes based on the whole set of clusters visited by the said block group.

Applying a similar ratio to the right hand side of the equation would initially yield $e^{Uial}/e^{Uiak}$, and taking a log of this ratio produces $U_{ial}-U_{iak}.$ Substituting in the utility functions from Supp. equation 3, we obtain a linear regression, where the dependent variable is the ratio of visiting probabilities between a specific cluster $k=1$ *a*nd the median cluster around it, and the independent variables are differences in travel costs, route utilities and cluster attributes:

$$ln(\frac{S_{i1}}{S_{ik}})=\alpha(T_{i1}-T_{ik})+\sum_{l=1}^{g} \mu_{l}(Z_{i1}-Z_{ik})$$

(Supp. Equation 4)

**References**

1. Statista. Number of smartphone users in the United States from 2018 to 2024 [Internet]. 2020. Available from: <https://www.statista.com/statistics/201182/forecast-of-smartphone-users-in-the-us/>.
2. Simpson EH. Measurement of Diversity. Nature. 1949;163:688.
