# Supplementary material for: The impact of COVID-19 on trips to urban amenities: Examining travel behavior changes in Somerville, MA
Source: PLoS One. 2021 Sep 1;16(9):e0252794. doi: 10.1371/journal.pone.0252794 (PMC8409662; doi:10.1371/journal.pone.0252794)
Supplement: Attachment — Submitted filename: REV1_Review Responses.docx [file pone.0252794.s004.docx]

Dear editor,

We greatly appreciate the opportunity to revise our manuscript, “The impact of COVID-19 on trips to urban amenities: Examining travel behavior changes in Somerville, MA.” The feedback provided has been valuable and offered a number of strong ideas for revising the manuscript.

We have revised the paper in several key ways. We have clarified our choice of the specific formulation of the discrete choice model we use for census block group aggregation; we have expanded the analysis to include all months throughout 2019 and 2020 to better understand the role of regulations and to more consistently track preference shifts over time; and descaled our model coefficients and presented them as distance equivalent coefficient ratios (or distance elasticities) which we believe lead to more intuitive and accessible interpretation of our results. These revisions have led us to revise all our figures and tables in the paper as well as some additions (new Figure 3a-3b; new Figure 4; new Table 3; new Supp. Table 4), which we think help provide better context and detail to the analysis. We have also more actively highlighted the limitations of the dataset, which, despite some shortcomings, provides a unique overview of amenity patronage at city-scale, offering insights into travel behavior for amenity-oriented trips that are not as widely researched as home-work journeys for instance.

We have also, more generally, revised the text to ensure it is as well-written as possible, while, per the request of the editor, revised the figures and updated all references to PLOS ONE style. We believe the paper has benefited greatly from this round of review. Please find below our point-by-point responses to the reviewers’ comments.

We can confirm that all the maps and figures in the paper (Fig. 1, 2, 6) are original. The basemap layers used in these images are all OpenStreetMap based, which allow CC BY 4.0 use.

Thank you.

**Editor**

- Discrete choice models using aggregate data are useful in many situations, which may be one of those situations. However, it would be best if you made this case clearly in the manuscript. I would like in your Analysis Framework section to do a better job motivating your empirical strategy, clearly explaining for a general reader why your method is valid to answer your research question. You describe your model in the appendix, but it is a general textbook-like description. I am looking more to explain why your empirical strategy is the best for your research questions, given the data restrictions.

We have added additional explanation in the Analysis Framework section to clarify why the particular type of discrete choice model we use is appropriate to use with aggregated data (pp. 15-16). We have also removed the sub-categorical analysis that was referring to differences in preferences across income and age groups from the paper, as we believe that this may have overstretched the potential of the model--there is unfortunately no way to observe whether particular trips were made by young, middle-aged, or elderly residents and making such associations based on CBG indicators risked venturing into ecological fallacy. We feel confident that the remaining block-group aggregations in the paper are justified and robust.

- You should also add caveats to your discussion about data limitations. These caveats are also recommended by Reviewer 1

We have added more clarifications about the limitations of the data as well as strategies we relied on to address these limitations, in the Discussion section, on pp. 35-36.

**Reviewer 1**

*Methods*

- How are the clusters determined? The methods do not describe this in enough detail. It sounds like the authors are familiar with the area. Did they know which businesses to cluster? Are there known boundaries of these clusters and the safegraph poi centroid is intersected with the known boundaries?

The clustering approach is explained in the data section on page 6. The clusters were formed by including only retail, food and beverage service, personal service and entertainment amenities, grouping them based on a minimum nearest-neighbor distance [100m] and minimum size of a cluster (10 amenities).

- Clustering visits may overestimate visits as the same device may enter multiple POIs in a cluster. Do you address this in any way? If not, this should be discussed in the limitations.

We have added further clarification to the limitations of the data and the model in the Discussion section on pp. 36.

- Why were the monthly aggregates chosen rather than the weekly patterns? Why were only certain months chosen when data is available from Jan 2019?

We originally examined trips at both fortnightly and monthly levels and eventually decided to run the analysis on a monthly basis due to significantly higher noise and variability in the fortnightly data. Our goal was to capture larger trends over time as opposed to weekly nuances. Based on your suggestion, we decided to include all months throughout the two-year period (i.e., Jan 2019 to Dec 2020).

- It sounds like trips were limited to only those from census block groups from within 2km. What is the rationale for 2km? How sensitive are the results to this choice? Were all other trips omitted from the dataset? Wouldn’t this impact the regression results if, for instance, trips from greater distances occur more frequently on the weekends?

The analysis only includes trips that are up to 3km in length. This includes over 85% of all trips at our study area clusters in a typical month in 2019. We limited the analysis because a) some limiting radius is needed in any case and a large share of visits captured within 3km seemed reasonable, and b) Somerville boasts of a high walking mode share with, for example, only 36% of individuals driving alone to work as compared to the national average of 76%. This suggested that a sizable proportion of trips would likely be arriving locally, on foot.

- The description of the empirical model should be in the main text and requires additional explanation. While this sounds like a standard model in some fields, PLOS One is interdisciplinary, and many won’t be familiar with the model. Equation 1 looks like a logistic regression, but the model description suggests that equation 4 is the one being estimated with OLS.

Indeed, the model we use is a specific linear formulation of discrete choice, shown in equations (1) and (2) and Supp. Equations 6. This formulation, originally dating back around to the 1960s, and more recently shown by Wheaton and DiPasquale (1996), Berry (1994) and Berry et al. (1995) makes it possible to estimate the model coefficients for aggregate groups (e.g., Census block groups). We have added more explanation about the specific formulation of this type of discrete choice model to pp. 15-16 and kept the more familiar formulation details in the Appendix.

- In either case, there are visits from the same CBGs going to different clusters. Wouldn’t that violate the iid assumption? It seems like clustering of standard errors is necessary, but without a better understanding of the model, I cannot recommend a specific structure.

Indeed, it would violate the iid assumption if we were estimating an ordinary linear regression model. However, the model we are using is a discrete choice model that compares various destinations (i.e., observed clusters) against a reference or baseline destination (i.e., a synthetic ‘median’ cluster). This model formulation has i.i.d. error terms but does not require clustering of standard errors. Please refer to the added explanation on pp. 15-16.

*Results*

- Table 1 results: Land value switches to a negative sign. This may be capturing the effects documented by Chetty et al. (2020) showing that higher-income areas are acting more risk-averse and likely have more capacity to avoid visiting stores.

Yes, this is an interesting point, and we have edited the text to highlight this on pp. 29.

- The distance results by income in Appendix T2 may be capturing sorting behavior. People with higher income may choose to live near the amenity clusters so as to avoid travel costs. Similarly, lower-income populations may not be able to afford to live near the amenity clusters and are forced to travel longer distances. Attributing this to preference seems misleading.

We have decided to eliminate the income and age based submodels in the revised paper, as we think this may overstretch the capabilities of the model. Due to the anonymized nature of SafeGraph data, there is no way of knowing that the trips made from lower age or lower income CBGs were actually made by lower age or lower income individuals. We think the grouping at the CBG level is consistent with the model assumptions and offers sufficient insight and material on its own.

- The authors provide no interpretation of the regression coefficients. Are they marginal effects? Is .3 large or small? The comparison over time is helpful but please provide an interpretation of the coefficients.

Thank you for this suggestion. We have substantially updated the analysis by showing the results for all months and transforming the resulting coefficients to ‘distance-equivalent’ coefficient ratios (or distance elasticities), which can be interpreted more readily and intuitively. (See updated pp. 19-21, a new Table 3 and updated results charts in Figures 5a-5c).

*Discussion*

- The first paragraph of the discussion claims that their model provides unique insights to policymakers about behavioral change and the effects of policies on visitation to inform programs to support businesses. Couldn’t we just look at the visitation data in real-time to measure how actual visitation is change and design compensation schemes to react to actual visitation? I believe the authors are trying to claim that they can produce counterfactual simulations with their model. This is an important point and contribution of the model. However, their model only implicitly captures COVID-19 risk by estimating the model in different months. Would including risk attributes that relate to COVID transmission enable further parsing of the effects? For example, could you calculate occupancy density a la Benzell et al. (2020) using the square footage and visitation information? Could you use variation in cases over time to help attribute behavioral changes to risk aversion? I recognize that this would raise endogeneity issues, but it would help address the benefits of suppressing the virus to businesses.

Integrating COVID19-specific variables into the model is an interesting prospect, but will have to remain the subject of future work. The model we have used for this analysis does not allow endogeneity in variables, e.g., using visitation data on the right hand side to measure occupancy density etc. However, our inclusion of the “percent large” variable is meant to examine how people’s preferences might have shifted vis a vis large indoor environments, where many people may be interacting in the same air environment. We reported on Figure 5b, Table 3, and pp. 28 that our model results indicate an aversion of clusters that contain a higher share of big-box stores in the early phases of the pandemic.

- The authors acknowledge in the discussion that partial closures or capacity limits are not explicitly considered in their analysis. The implication of this omission is that policy/regulation is mistaken for behavioral change. Have these regulations changed over time in Somerville? If so, you may be able to exploit this variation to parse out how much is regulation versus consumer choice.

This is an interesting suggestion too. We believe it would be too difficult here to capture variations in partial reopenings since not only have stores resorted to take-outs or curb-side pickups during some of the shut-down order periods, but many have voluntarily continued that practice and changed their opening hours, which we unfortunately do not have data on. We acknowledge these shortcomings in the discussion, but also think that adding more months in the revised paper can help us get a better sense of regulatory effects and behavior changes over the year.

- How generalizable are the results? The study area is in close proximity to several universities. I would assume the area is relatively high income and high education.

While the reviewer’s point is taken, there is a good deal of variability in incomes and demographics across the 163 Census block groups we include in the model. Particularly those in Somerville and Medford introduce more lower-income populations to the study area than those in the parts of Cambridge and Boston tha the model covers. We can not quantify how generalizable the results are from our data, but the effects we find seem consistent with our intuition and could be similar elsewhere. That being said, extending this analysis to other areas in the country would be an appropriate next step for future research.

*Presentation*

- The variable labels in Table 1 are hard to read (they look like code names). Can you convert them to something more readable?

We have updated the variable names for more clarity in Table 1, Table 2a-2b, Table 3, Figs 5a-5c.

- The graphical presentation of the results is an effective visual for communicating the results. However, there is no indication of estimate uncertainty (other than the p-value significance). 95% confidence intervals on the estimates would convey the information in the graphic. The points could be slightly shifted left and right so the plot elements do not overlap. The lines connecting the point estimates could be removed to minimize clutter since they do not convey much information. Along these lines, the authors ask the reader to visually compare the results across time, but do not provide a statistical comparison. The standard hypothesis tests reported in the table pose a null hypothesis that the coefficient is 0. We are interested in how the coefficient differs over time (relative to last year). The authors choose to estimate the models for each year-month separately, which is fine. Estimating them in a single model with dummy variables would provide the coefficients (and hypothesis tests) that would address my comment. Standard errors should certainly be clustered by year-month in that case.

Using the updated ‘distance-equivalent’ coefficients (or distance elasticities) allows us to compare variable effects over time. Additionally, we now include confidence bands that inform whether the impacts in a particular year are statistically different from the impacts in the previous year. Our comparison suggests that although the mean values have changed, most coefficients, except Num_Establishment, Cluster Area, % Large and Cluster Diversity, have not changed over time if we consider the 95% confidence interval for inferring statistical significance. We have also updated the discussion about this in the paper’s Results and Discussion sections.

- Report standard errors rather than p-values in Table 1.

We have updated the results tables accordingly.

- Line 870: I believe mean should be median.

Yes, updated, thank you.

- Line 524 describes the upper left panel of Fig 6 as comparing April visits in 2020 to 2019. However, the figure indicates a comparison of observed trips to expected. Is expected a model prediction? If so, I think the text needs to be changed. If not, it seems out of place in this figure.

We have called this expected (reflecting what the expected trip count would be sans COVID-19), since we adjusted the expected year-on-year change based on February differences between 2019 and 2020. So we are not comparing the observed 2020 April numbers directly against April 2019 numbers, but also adjust the comparison to expected annual difference between the periods. We include a note about this in the caption of Figure 6 and have further clarified the text.

- Figure 6 components should be labeled a-d, and referenced in the text. The color scale is misleading. I suggest a divergent color scale with a neutral color at 0% change, so increases are clearly different from decreases.

We have followed your suggestions in the updated Figure and text on pp. 33-34, thank you.

- The left two panels of figure 6 seem to fit better with the section describing the data. They do not depend on the estimated model like the right two panels.
  - We have included a separate new Figure 3b, which illustrates the annual visit differences between clusters spatially in the Analysis Framework sections. While the two left figures in Figure 6 are indeed based on observed data (with an annual adjustment as noted above), we have decided to keep them next to the predictions so that the readers can observe the change more readily, without having to flip through different parts of the paper.

**Reviewer 2**

*General*

- The study objective is to examine the changes of travel behavior, as the title, abstract, introduction emphasize, however, this paper actually focuses on the change of the visit - the customer flows - to these business clusters. The authors describe “… such as aggregated visit count as well as dwell time spent in various destinations” in their Data section, suggesting that the data from the SafeGraph only can provide the visit data, rather than the individual-level behavioral data, which largely confuses the readers about the research subjective of the study. If focusing on behavioral changes, collecting the data for each resident is necessary, for example, if the dataset provides a unique GPS_User_ID, then using the ids to track their shopping and other business activities behaviors in 2019 and 2020, before and after restrictions, are appropriate to answer the research questions. However, if using the aggregated flow data, how to distinguish random visitors to these destinations from the regular customers? The whole paper emphasizes many times about the OD, trip behavior choice and changes, impacts on future trip visits to these destinations, which strongly implies the study objective should be the individual level behavior changes and their influences on the aggregated level visit numbers to the same destinations. Thus, a more appropriate use of the aggregated flow data to the business should be developing a model to predict the future visits considering the impacts of the COVID-19. However, using year 2019 data only will be a limiting point in this direction, since the prediction accuracy will be largely affected by the amount of historical data. Going this research direction will need to collect more historical data and put the 2020 shutdowns/restrictions as factors to the model to predict future flow changes. If the authors want to predict individual behavior changes based on their current data, they can find the user_id to re-process their data and do the prediction. Currently, machine learning models or deep learning models can help to do a good job, for example, Zhu et al., 2017. Then, they can stick to their research questions about evaluating the systematic impacts on these business destination clusters based on the residents’ behavior changes. Of course, they can consider the random factors from tourism visitors to these destinations to make the future prediction model more accurate.
  - While we believe that the study that the reviewer proposes sounds interesting, we only have access to anonymous and aggregated trip counts from Census block groups to individual amenities (which we group at the cluster level). SafeGraph does not publicize individual user IDs, even if anonymized for privacy reasons. The model we use was originally designed to perform this type of group-level choice analysis and we believe the anonymized O-D flow data from SafeGraph offers an innovative case for such a model.
  - Doing more historical trend analysis and future forecasting about cluster visits post-pandemic offers interesting ideas for future research. We will certainly consider it for follow-up work, but this remains beyond the scope of this paper, which is aimed at understanding how both trip volumes and destination preferences have shifted during COVID-19 as compared to the year prior.
  - We have expanded the text in this revision to explain our choice of the discrete choice modeling framework as well as the interpretations of the results that emerge. As mentioned above, in response to another reviewer, we have eliminated subgroup level analyses (income and age groups) due to the limitations of our data.

*Modeling/Data*

- The modeling section: nowadays, behavioral models are more suitable to analyze travel behavior changes, rather than discrete models. Since point 1 already describes some main concerns regarding the dataset, and the feasibility of using such data to answer certain questions. I recommend the authors take a deep examination of the GPS dataset (as well as the built environment data, policy change data, e.g., the widely use and provision of the masks in a lot of businesses, which might be helpful in the prediction), so that to have a better understanding of the data limits, characteristics, and research capacities.

We have added more discussion about the model suitability on pp. 15-16 and on data limitations to the end of the paper, on pp. 35-36. Furthermore, we have revised how we interpret the coefficients in this revision, now relying on intuitively comparable coefficient ratios (or elasticities) instead of raw coefficients in the Results section.

- Also, a detailed analysis of the demographic and economic characteristic of the GPS data population is needed, especially the study area is not a big area, such as multiple cities or the whole nation. If only simply describe - everyone nowadays has a phone so they are captured by the SafeGraph company - should not be acceptable. A more rigorous analysis of the data’s representativeness is very essential in this direction of research on localized analysis in particular.

The reviewer raises an important point that cell phone trace data is not fully representative of all demographics. Nonetheless, the city that we selected has a population with characteristics that are likely to result in high penetration rates. While appropriate data are not available to detail SafeGraph penetration in specific geographies, we have addressed this issue within the text on pp. 5-6 and in Supp. Materials B.
