# Supplementary material for: The impact of COVID-19 on trips to urban amenities: Examining travel behavior changes in Somerville, MA
Source: PLoS One. 2021 Sep 1;16(9):e0252794. doi: 10.1371/journal.pone.0252794 (PMC8409662; doi:10.1371/journal.pone.0252794)
Supplement: Attachment — Submitted filename: REV2_Review Responses.docx [file pone.0252794.s005.docx]

Dear Editor,

Thank you for the response to our revised manuscript, “The impact of COVID-19 on trips to urban amenities: Examining travel behavior changes in Somerville, MA.” Please find our answers to the only comment we received from Reviewer #1 below. We hope this adequately addresses all the questions raised.

**Reviewer 1**

[1] I like the new distance elasticities as a normalization to compare across time. However, the text does not describe how standard errors are calculated (e.g., delta method?, bootstrap?), which impacts inference.

Standard errors were calculated using the delta method, which has been found to be appropriate for use with large samples and is most accurate for well-conditioned data (Hole, 2007). Since our sample size is relatively large [n (2019): min=509; max=665. n (2020): min=171; max= 568] and well-conditioned, we opted to use the delta method. Hole (2007) also reports that both the delta method and bootstrapping produce fairly similar confidence intervals in empirical evaluations of willingness-to-pay measures (which are theoretically equivalent to our distance elasticity measures). We have added a note about that on page 13, as well as the Hole (2007) reference below.

Hole, A. R. (2007). A comparison of approaches to estimating confidence intervals for willingness to pay measures. Health economics, 16(8), 827-840.

[2] The authors provide an improved description of the model. One of the key insights of the BLP model is that price is endogenous because of the unobservables in the error term. The authors use distance as a proxy for travel cost (price). You might add an explanation for why your travel cost version of the model is not subject to the same criticism. Or, if it still may be, how you would expect it to affect the estimates (if bias, which direction).

We believe that our model may not be subject to the same criticism because, unlike the BLP model, the unobservables in the error term are not due to a measurement error in travel distance (price). The travel distance between any two block groups is known a-priori and is deterministic. We compute network distances (using road networks from OpenStreetMaps) as a measure of travel distance, instead of the computationally faster but less realistic Euclidian distances.

Admittedly, if we were to use travel time, there could be measurement errors in travel time based on the travel mode. However, that would lead to a more sophisticated model structure where we would want to explore destination choice by travel mode, e.g., a nested or joint choice of both destination and mode (Jonnalagadda et al., 2001). Our data do not provide information on travel mode, so we are limited to infer only destination choice preferences.

Jonnalagadda, N., Freedman, J., Davidson, W. A., & Hunt, J. D. (2001). Development of microsimulation activity-based model for San Francisco: destination and mode choice models. Transportation Research Record, 1777(1), 25-35.
